# Insomnia, Short Sleep, and Their Treatments: Review of Their Associations with Weight

**DOI:** 10.1007/s13679-024-00570-3

**Published:** 2024-05-22

**Authors:** Kelly C. Allison, Lindsey Parnarouskis, Molly D. Moore, Alyssa M. Minnick

**Affiliations:** 1 Center for Weight and Eating Disorders, Department of Psychiatry, Perelman School of Medicine, University of Pennsylvania, 3535 Market Street, Suite 3029, Philadelphia, PA 19104-3309 USA; 2 Department of Psychology, University of Michigan, Ann Arbor, MI 48109 USA; 3 InBody BWA, Audubon, PA 19403 USA

**Keywords:** Insomnia, Short sleep, Sleep extension, Hypnotics, Weight, Obesity

## Abstract

**Purpose of Review:**

Insomnia and short sleep have been linked with weight gain and obesity. However, these findings have not been consistent across studies. We review recent evidence for the association between insomnia, short sleep, and weight gain, as well as the relationship between behavioral and pharmacological treatments for sleep and weight.

**Recent Findings:**

The relationship between insomnia and obesity is mixed, with stronger associations between insomnia with short sleep and obesity than other presentations of insomnia. Short sleep is associated with weight gain. Z-drugs and benzodiazapines do not appear to impact weight, but many antidepressants and antipsychotics that are used for insomnia treatment do cause weight gain.

**Summary:**

The relationships between insomnia and short sleep with weight gain and obesity are inconsistent. More prospective trials are needed to identify mediators and moderators of this relationship to better develop and deliver effective interventions for both sleep and weight problems.

## Introduction

Insomnia and short sleep provide increased opportunity to eat and higher energy needs that can be overcompensated by individuals, and, therefore, may be linked to obesity. Insomnia is the most prevalent sleep disorder, affecting approximately 10% of the population. It is defined as difficulty initiating and maintaining sleep and/or waking earlier than intended with the inability to fall back asleep at least three nights/week, for a period of at least three months [1–3].

Short sleep can manifest with insomnia, but it often is a consequence of lifestyle factors. The American Academy of Sleep Medicine defines short sleep as < 12 h for children aged 4–11 months, < 11 h for children aged 1–2 years, < 10 h for children aged 3–5 years, < 9 h for children aged 6–12 years, < 8 h for adolescents aged 13–17 years, and ≤ 6 h of sleep/day for adults [4, 5]. Recent estimates suggest short sleep affects approximately 21–25% of adults and 35% of children and adolescents [6, 7].

Treatments for insomnia and short sleep may also impact weight. It is unclear how insomnia may impact the efficacy of weight loss treatments, or if insomnia treatments might improve weight management, including prevention of weight gain or producing weight loss in those with obesity. Further, many pharmacotherapies for insomnia and its comorbid psychiatric disorders are associated with weight gain and metabolic dysfunction. We conducted a narrative review of these topics, focusing on the past five years.

## The Relationship Between Insomnia and Weight

It is well established that overweight and obesity are linked to obstructive sleep apnea [8]; however, the connection between obesity and insomnia is less clear. Insomnia impairs daytime functioning and is associated with excess eating through various mechanisms, including shortening the sleep period which is related to increased ghrelin and lowered leptin, overcompensation for higher energy requirements with longer waking hours, longer time available to eat, increased higher fat/higher calorie food consumption, and lowered physical activity due to fatigue [9, 10]. However, the research surrounding insomnia’s connection to weight, and more specifically, obesity, is inconclusive [11–13].

### Childhood and Adolescent Studies

A small amount of evidence has connected weight and insomnia in youth. Specifically, in adolescents, body mass index (BMI) z-score was positively associated with insomnia severity, but not in younger children [14]. Furthermore, improvements in insomnia over time were not linked to BMI in children or adolescents [14].

### Adult Studies

A handful of studies have found no association between insomnia and BMI [15] or between insomnia and obesity [16]. However, other studies have found that insomnia is linked to *lower* weight or BMI [17–19]. Furthermore, a meta-analysis [20•] revealed that an insomnia diagnosis did not significantly increase the risk of obesity as compared to those without a diagnosis (*OR* = .80, *p* = .61). However, insomnia symptoms and BMI showed a small correlation (*r* = .06, *p* = .03) [20•].

To find mediators between insomnia and obesity, van Buuren and Hinnen [16] investigated socioeconomic status (SES) and other influences such as depression, self-control, emotional regulation, and worry that seem to underlie both conditions. The authors found little association between these factors, insomnia and obesity, indicating that the two diseases are not overlapping health conditions [16].

In contrast, some literature established a direct association between weight and insomnia. A meta-analysis [21] showed that individuals with insomnia had a higher risk for obesity compared to individuals without insomnia (*OR* = 1.31). Insomnia symptoms were also associated with high BMI in a subsequent cross-sectional study [22]. Further, one longitudinal study found that weight gain over a decade increased risk for developing insomnia (*OR* = 2.78) [23]. Interestingly, no relationship was found between weight at baseline and development of sleep issues over the course of the study [23]. However, another prospective study linked obesity at baseline with increased risk of developing chronic insomnia [24], and women with persistent insomnia through two years postpartum were more likely to gain weight than those without insomnia [25].

### Insomnia and Weight: Potential Mechanisms

**Fig. 1:**
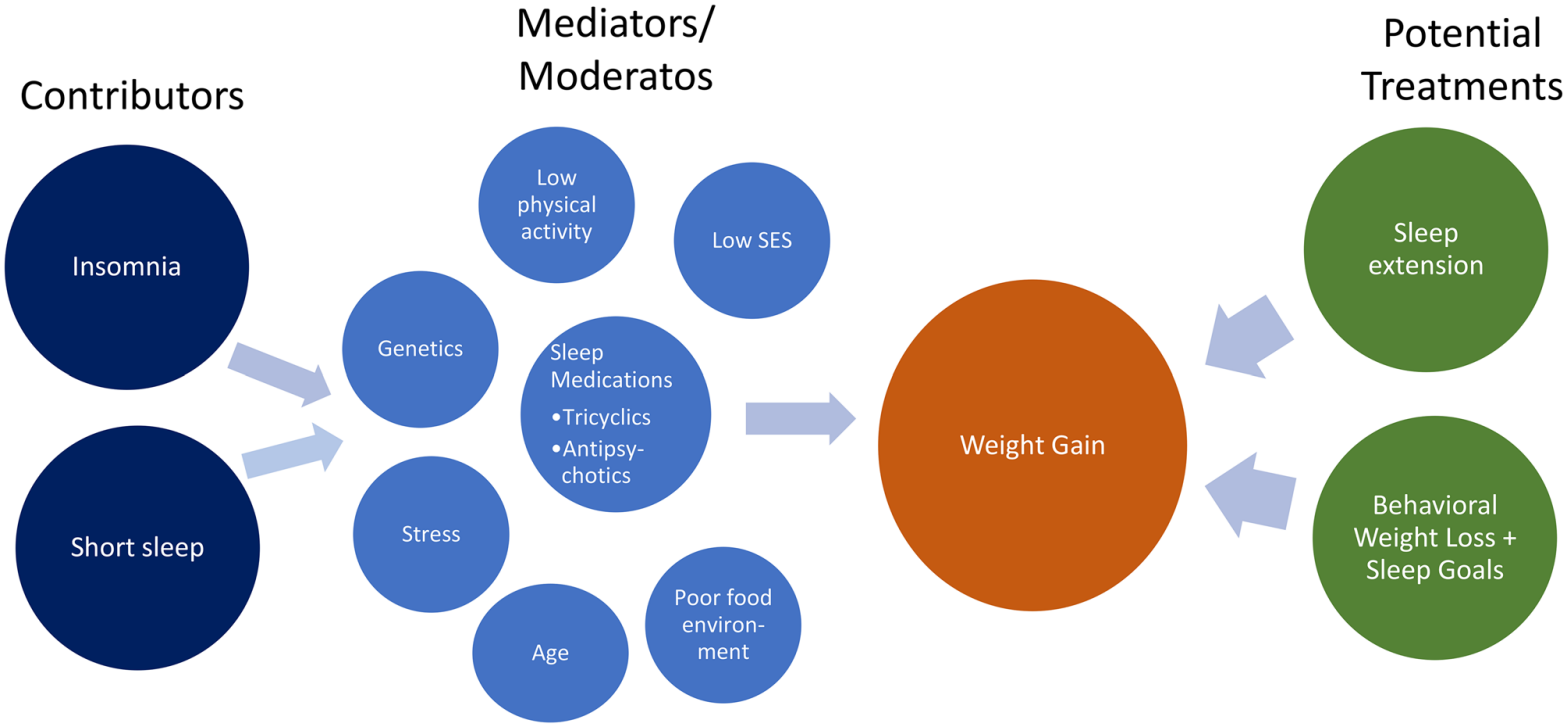
Hypothesized sleep-related contributors to weight gain

Inconclusive evidence that insomnia and obesity are related has limited research on potential mechanisms explaining this link. Most research on this topic is cross-sectional and focuses on sleep difficulties (not specific to insomnia), making it difficult to determine mechanisms and causality. However, some literature speculates on possible behavioral, environmental, psychological, and genetic mechanisms. Figure 1 outlines the various factors that could contribute to the relationship between insomnia and obesity based on recent literature.

Behavioral factors may influence the relationship between insomnia and obesity. Decreased energy expenditure and a sedentary lifestyle are contributing factors for obesity [26]. Physical activity has also been linked to improved subjective sleep quality and insomnia severity [27, 28]. In a longitudinal study, exercise improved insomnia symptoms in men with BMI > 25 compared to those with healthy weight, although body weight did not decrease over the 6-month intervention [28]. In adolescents, technology use (i.e., screen time) can be a risk for both obesity and poor sleep [14]. With the clear link between increased physical activity and improved insomnia symptoms, there could potentially be a link between lack of physical activity, weight, and an insomnia diagnosis.

Psychological distress is associated with insomnia as one of the causes for difficulty with falling asleep as well as being the result of insufficient sleep [2, 29]. In adolescents, insomnia mediated the relationship between psychological distress and weight [30]. There may also be a genetic link between insomnia and obesity, which may identify phenotypes for those with obesity or insomnia at risk for developing the other condition [31, 32].

Despite these potential mechanisms explaining the loose connection between insomnia and weight, much of this research is cross-sectional. More longitudinal research is needed to establish the connection between these two health conditions to establish causality, and if the connection is apparent, establish mechanisms that lead to their overlapping risk factors.

### Insomnia with Short Sleep

Research has emphasized the need to distinguish between insomnia with and without short sleep [11, 33, 34] and has demonstrated that insomnia with short sleep is more clearly linked to obesity [22, 35, 36•]. Perhaps it is short sleep duration specifically that is an important mechanism explaining the relationship between weight status and insomnia.

## The Relationship Between Short Sleep and Weight

### Short Sleep and Weight Gain Among Children and Adolescents

Several studies found that short sleep was associated with greater incidence of overweight and obesity among children and adolescents [37–39]. A large cross-sectional study of Chinese children (*N* = 35,414) found short sleep duration (by age group) was associated with 1.24 increased odds of obesity (CI: 1.14–1.35) and very short sleep (< 7 h/day for ages 6–13 and < 6 h/day for ages 14–17) was associated with 3.01 greater odds of obesity (CI: 2.19–4.15) [37]. Long sleep duration (> 11 h/day for ages 6–13 and > 10 h/day for ages 14–17) was not associated with overweight/obesity. Another study of Chinese children and adolescents (*N* = 2019) found that short sleep duration was associated with significantly greater odds of overweight or obesity (OR = 1.32, CI: 1.06–1.64) compared to recommended sleep duration, independent of self-reported sleep quality [38]. However, this relationship may not be uniform across childhood and adolescence. For example, when Chen and colleagues [38] stratified their sample by age group, short sleep duration was associated with increased odds of overweight/obesity among 8-13-year-olds (OR = 1.34, CI: 1.05–1.71), but not among 14-16-year-olds, which is opposite of the findings for these age groups for the association between obesity and insomnia [40].

A large study of Korean adolescents (*N* = 22,906) found both short sleep duration and short weekend catch-up sleep (CUS) duration were associated with obesity status [39]. BMI was inversely related to average sleep duration and weekend CUS duration, suggesting CUS during weekends may mitigate the effects of short sleep during the week. This contrasts with prior findings suggesting greater sleep duration variability is associated with greater incidence of overweight/obesity [41].

A nationally representative study of Korean girls (*N* = 303) found that girls reporting recommended sleep durations had significantly lower whole-body total mass as measured by dual-energy x-ray absorptiometry (DXA; 46.5 kg vs. 51.4 kg), fat mass (14.4 kg vs. 17.6 kg), and fat mass percentage (30.7% vs. 33.2%) compared to girls reporting very short sleep (< 7 h among 6-17-year-olds) [42]. Sleep duration was also negatively associated with DXA-assessed total mass, fat mass, and fat mass percentage across various body regions.

Other studies found no associations between sleep duration and adiposity, perhaps due to small sample sizes [43, 44]. A study of 125 U.S. children aged 8–17 years found that later bedtimes on weekdays and weekends were associated with higher systolic blood pressure [43]. However, sleep duration and bedtimes were not associated with other components of metabolic syndrome or body composition. Another small study of U.S. children and adolescents (*N* = 123) found no associations between actigraphy-assessed sleep duration and DXA-assessed adiposity [44].

Further, a study of U.S. children and adolescents in two age groups (5–11 and 12–18 years; *N* = 59) examined basal metabolic rate (BMR), total energy expenditure, and physical activity in addition to sleep actigraphy and DXA [45•]. They found that sleep duration accounted for variance in BMR (β = 0.11) but not total energy expenditure. Among 5-11-year-olds, children meeting their sleep recommendations at least half of seven days had more light physical activity and less sedentary time compared to children meeting these recommendations less often, suggesting short sleep may contribute to weight gain through decreased BMR and physical activity.

Three studies illuminated potential sociodemographic differences in the relationship between sleep duration and weight [46–48]. A secondary analysis of 24,000 U.S. adolescents found that the association between longer sleep duration and decreased odds of overweight/obesity only applied to White participants, participants with family income > 400% of the poverty level, and participants whose primary caregiver had a high school degree or higher [47]. Hispanic adolescents and those with household income below the poverty line, and caregivers below high school education showed weakened but reversed associations – longer sleep was associated with increased odds of overweight/obesity. Although this finding did not reach statistical significance, it points to potential ethnic, educational, and socio-economic differences that should be explored further. Additionally, a cross-sectional study of New Caledonian adolescents found short sleep duration was associated with increased overweight/obesity for European adolescent, but not ONENA (Oceanians of Non-European, Non-Asian descent) adolescents [46]. Thus, the association between short sleep duration and weight gain may be more salient for European/European-American individuals.

A study of Canadian adolescents (*N* = 4,991) examined sex and sleep duration as moderators of the relationship between social media use and BMI [48]. Sleep duration mediated the relationship between social media use and BMI (β = 0.004, CI: 0.001–0.008) among males but not females. Although this study did not examine a direct association between short sleep and BMI, the findings point to potentially important sex differences in the role of social media and sleep duration in weight outcomes.

### Short Sleep and Weight Gain Among Adults

Several studies provide support for the association between short sleep and weight gain among adults. An analysis of participants aged 18–59 from the US NHANES (*N* = 5151) found a negative association between sleep duration and DXA-measured visceral fat mass for both men and women [49•]. A cross-sectional study of healthcare workers in Brazil (*N* = 200) found night shift workers had higher prevalence of short sleep duration, higher weight, BMI, and abdominal circumference [50]. A cross-sectional study of U.S. undergraduate students *(N* = 307) found that shorter average nightly sleep duration was associated with higher BMI [51]. However, total sleep duration was not associated with differences in BMI, which suggests napping cannot overcome the effects of short nightly sleep duration on weight.

A cross-sectional study of Moroccan undergraduates (*N* = 438) found that both short and long sleep duration were associated with overweight/obesity among men [52]. This finding is consistent with prior research suggesting that individuals both above and below the recommended amount of sleep experience increased weight gain and related metabolic abnormalities, and that this relationship may be stronger for men [53].

One eight-year longitudinal study following Chinese adults (*N* = 21,958) found that individuals who self-reported short sleep duration were at higher risk for significant weight gain (≥ 5 kg) than those reporting sleeping seven hours (OR = 1.13, CI: 1.02–1.29) [54]. Additionally, short sleep was associated with central obesity, but not general obesity (OR = 1.13, CI: 1.00–1.28). The association between short sleep and weight gain was also stronger for individuals who were physically inactive at baseline. These findings suggest that sleep duration may impact visceral fat more than subcutaneous fat, which may be moderated by physical activity.

A study of Swiss adults aged 35–75 years (*N* = 2162) found that short sleep was not associated with clinically significant weight gain (≥ 5 kg) over 5 years [55]. However, poorer subjective sleep quality, greater percentage of stage 2 sleep, lower oxygen saturation during sleep, and greater autonomic arousal duration were associated with increased odds of clinically significant weight gain over time. Thus, sleep quality may be more important for weight outcomes than sleep duration per se, particularly among middle-aged to older adults.

### Summary

Recent studies continue to support previous findings that the magnitude of associations between short sleep duration and weight gain appears to be stronger among children and adolescents than adults. Ethnic, SES, and educational differences are worth further exploration as moderators, particularly among children/adolescents. Studies conducted with adults suggest mixed findings regarding short and long sleep and associations with BMI, visceral adiposity, and metabolic syndrome. Greater variability, later sleep timing, and poorer sleep quality may be more important for driving weight and metabolic outcomes among adults. Future research should also consider sex differences in the association between sleep duration and weight gain.

### Behavioral Intervention Studies for Insomnia and Short Sleep: Association with Weight

No studies to date have examined the impact of insomnia treatment, such as cognitive behavioral therapy for insomnia (CBT-I), on weight loss. Most research in this area focuses on sleep hygiene and related strategies to improve sleep in the context of weight loss. Sleep extension shows promise as a sleep-focused intervention that may influence weight outcomes.

### Infant, Child, and Adolescent Studies

Interventions start as early as the prenatal and infancy periods to improve sleep, eating, and physical activity behaviors to prevent childhood obesity. Taylor and colleagues [56] randomized 802 pregnant women to one of four groups in a community-based clinical trial: (1) usual care control group; (2) intervention to improve food, activity, and breastfeeding (FAB); (3) sleep (behavioral strategies to improve infant sleep); and (4) combination (FAB and sleep). At 24 months postpartum, there were no significant group differences in nighttime sleep duration for children and no significant effects on BMI. There was, however, a lower prevalence of obesity in the sleep-only and combination groups (19% and 21%, respectively) than the FAB and control groups (40% and 33%, respectively) [56]. It was unclear how the sleep intervention influenced obesity given that sleep duration did not differ between groups, so future studies should explore the mechanisms of action in early life.

Childhood studies provide evidence for the positive impact of sleep extension on body weight. Hart and colleagues [57] tested sleep extension interventions in 8–11-year-old children, initially finding decreased motivation for food in the sleep extension group in parallel with a 40-minute increase in nighttime sleep as compared to the control group who showed a 16-minute decrease in nighttime sleep, providing a signal that sleep extension may impact eating behaviors. In a larger study (*N* = 37) [58], children completed a 3-week within-subjects, counterbalanced, crossover design that included one-week each of habitual sleep, 1.5 h/night of increased time in bed (TIB), and 1.5 h/night of decreased TIB. Body weight and daily caloric intake decreased (-0.22 kg and -134 calories, respectively) during the sleep extension week versus the reduced sleep week. In a subsequent, larger efficacy trial, the intervention group experienced +40 min of nighttime sleep and went to bed 37 min earlier than the control group. There were no significant group differences in body weight or daily caloric intake after 2 months; however, post-hoc analyses indicated lower BMI and fewer calories consumed from fat among children who extended their sleep by 30+ minutes/night (across groups) [59••].

Among adolescents in South Korea, researchers had a unique opportunity to assess body weight following a policy change in three of 16 regions which closed tutoring centers early, surveying 191,799 adolescents (7th–12th grades) between 2009–2012 [60]. The policy change resulted in an additional 17 min of nighttime sleep; the control regions had no changes in sleep. For every 1 h increase in sleep duration, there was a 1.6 kg reduction in weight and a 4.2% reduction in the probability of having overweight/obesity.

In another study, Moreno-Frias and colleagues [61] instructed 52 adolescents to follow a 500-calorie/day deficit diet plan for 4 weeks with or without sleep extension. The intervention group extended their nighttime sleep by 1.2 h/night compared to a 30 min/night increase in the control group. The sleep extension group lost more weight (*M* = -2.1 kg *±* 1.6, *d* = 1.33) than the habitual sleep control group (*M* = -1.2 kg *±* 1.6, *d* = 0.69) [61]. Although change in body weight was small, it is possible that sleep extension may improve health at the population level with decreased prevalence in overweight/obesity.

### Combining Behavioral Weight Loss and Sleep Health among Adults

Studies have also examined interventions among adults that incorporate sleep goals into behavioral weight loss programs. Two early, pilot studies examined sleep enhancement interventions coupled with behavioral weight loss. One study (*N* = 49) incorporated sleep counseling after week 4 in a weight loss treatment [62], and another (*N* = 25) offered sleep strategies prior to weight loss [63]. In the first study, the group that integrated sleep strategies lost significantly more weight (5%) at post-intervention than the weight loss only group (2%) [63]. In both groups, perceived stress decreased and interestingly, sleep efficiency improved. In the second study, however, objectively-measured nighttime sleep duration did not significantly change during the first 4 weeks in the sleep or control group. The sleep plus weight loss group lost significantly *less* weight than the weight loss plus health education group (-2.5 *±* 2.5% vs. -4.8 *±* 2.6%, respectively) at 6 weeks, with no significant group differences at post-treatment (routine: -3.4 *±* 4.7% vs. education: -6.4 *±* 6.0%) [63].

More recent studies have used technology-based interventions. One conducted a 3-arm, 6-month, parallel-group randomized clinical trial (*N* = 116) with a 12-month follow-up that included: (1) traditional diet and physical activity weight loss group, (2) enhanced group targeting diet, physical activity, and sleep, and (3) waitlist control group [64]. The interventions consisted of one in-person session, then ongoing smartphone app use with calorie counting and tracking devices (e.g., Fitbit, scale, etc.). The enhanced intervention group received sleep psychoeducation, sleep hygiene, stress management, cognitive behavioral self-regulation strategies, and weekly personalized feedback. Insomnia symptom severity was significantly lower at 12 months in the pooled intervention group compared to the control group; however, bedtime variability did not differ between groups. Primary results indicated no significant differences between the control and pooled intervention groups on weight loss at 6 months [group difference, 95% CI: -0.92 kg (-3.33, 1.48)] and 12 months [group difference, 95% CI: -0.00 kg (-2.62, 2.62)] [65]. Of note, this trial included a homogenous sample of 71% women and 94% White participants. Attrition was high (70% retention at 6 months and 47% at 12 months).

Another study tested the feasibility and acceptability of a text-messaging based intervention among emerging adults (18–21 years, *N =* 43) with overweight [65]. It compared diet and physical activity vs. diet, physical activity, and sleep (SLEEP) interventions. Both provided an initial in-person visit to set behavioral goals and subsequently generated tailored text messages for 3 months. The SLEEP group extended sleep by 1 h/night, whereas the standard group had no significant change. Despite differences in sleep, there were no significant reductions in BMI. BMI remained stable through 6-months, and the authors indicated these findings may be important given that weight gain is common during college [66].

Two other studies focused on special populations. Hoerster and colleagues [67] developed a tailored behavioral weight loss intervention for veterans with PTSD. Although not focused exclusively on sleep goals within the Veteran Affair’s MOVE behavioral weight loss program, a sleep assessment and “improvement plan” was included in one session. Following the 16-week program with 8 participants, 71% had clinically meaningful weight loss (*≥* 5% of baseline weight), with an average loss of 6.1% (*SD* = 2.1). PTSD and insomnia symptom severity also improved at post-treatment [67]. Another study tested a 16-week multi-component intervention (nutrition, sleep, and physical activity) vs. waitlist control among airline pilots with overweight (*N* = 125; 9.6% women) [68]. At treatment end, the intervention group increased their nighttime sleep [*M* = 0.7 h/day (0.6–0.8)] more than the control group [*M* = 0.1 h/day (0.0–0.2)]. Intervention participants also showed a significantly larger reduction in weight (*M* = -5.5 kg, 95% CI: 4.8–6.1) than the control group (*M* = 0.4 kg, 95% CI: 0.1–0.7).

In summary, there are mixed results across interventions that integrate weight and sleep goals. These studies have been limited by small samples and/or demographic homogeneity of participants. Future studies should focus on mechanisms by which sleep impacts weight to determine if additional treatment targets are needed. Intervention delivery should also be considered to determine if technology-based programs provide the appropriate level of support to facilitate long-term health behavior change.

### Sleep Extension Interventions for Adults

Sleep extension is a stand-alone behavioral treatment that has been considered for weight management. The goal is to extend nighttime sleep duration by at least 30–60 min/night with behavioral sleep and behavior change strategies [69]. Sleep extension has been studied as a treatment to improve multiple outcomes, including eating behaviors, weight, and cardiometabolic health. To-date, only six sleep extension studies have reported on weight loss outcomes, although none focused on body weight as a primary outcome. Of note, study samples were relatively homogenous, with most studies including a majority White women [70–72] and two studies that did not report participant race/ethnicity [73, 74]. Five studies did not find significant weight changes after sleep extension, some in comparison to a habitual sleep control group [70–74]. These studies only extended nighttime sleep by 21–49 min/night, so the effects on weight were likely limited [70–74]. The final study found a slight reduction in weight among the intervention (*M* = -0.48 kg, 95% CI: -0.85, -0.11) vs. control (*M* = +0.39 kg, 95% CI: 0.02, 0.76) groups across 4 weeks, two weeks of which included the sleep extension intervention [75••]. The intervention group extended their nighttime sleep by an average of 1.2 h/night compared to the control group, which may have contributed to reduced opportunity to eat, lower appetite and food cravings [76], and possibly improved energy for physical activity.

### Summary

Among sleep extension-only studies, few assess weight change. Of these, only one produced significant weight loss [75••]. Notably, this growing body of literature is limited by short-term study durations (up to 6 weeks of active intervention) [70, 71, 73], heterogeneity of treatment protocols, and homogeneity of participant samples. Future studies should develop a standardized sleep extension treatment protocol and use longer study periods given that at least 6 weeks are required to achieve clinically meaningful weight loss; long-term follow-up remains important.

## Pharmacotherapy for Insomnia: Association with Weight

One of the most consistent links between insomnia and increased weight results from use of pharmacotherapies for insomnia.

### Epidemiological and Association Studies

Two prospective Swiss cohorts comprised of 2861 patients with psychiatric disorders were examined together for associations between an insomnia diagnosis or prescription of sedative medications and weight change over time [77]. Thirty percent had insomnia disorders, and those participants tended to be older (45 compared to 39 years old) and more likely to identify as female (53% compared to 46%) than those without insomnia disorders. Those with insomnia disorders were more likely to be prescribed psychotropic medications with high risk of weight gain (24% vs. 20%, respectively), including olanzapine, clozapine, and valproate, as well as a higher chance of having a diagnosis of schizoaffective or bipolar disorders. Further, being prescribed these medications was associated with double the risk of having an insomnia disorder. BMI, waist circumference, and fasting glucose were all higher in those with insomnia disorders compared to those without, as well as higher prevalences for central obesity (58% vs. 52%) and metabolic syndrome (25% vs. 17%), respectively.

### Hypnotics and Weight

Benzodiazapine receptor agonists and “Z-drugs,” including eszipiclone/zopiclone, zaleplon, and zolpidem, are the most prescribed medications for insomnia. There is little evidence that these medications are associated with weight change. Unfortunately, weight change outcomes are not generally included with treatment or adverse event data for this drug class [78, 79]. Continued efforts should be made to standardize the reporting of weight change for all pharmacotherapies to better inform patients.

### Antidepressants for Treatment of Insomnia: Relation to Weight

Many antidepressants interrupt sleep through their action on 5-HTP (serotonergic) receptors and by increasing noradrenaline and dopamine transmission. These include SRNI, NRIs, MAOIs, SSRIs, and activating tricyclics. However, some antidepressants (sedating tricyclics, trazadone, mirtazapine, and nefazodone) antagonize 5HT2 receptors, producing an antihistamine action and promoting sleep [80]. Doxepin, a tricyclic, is the only FDA approved antidepressant for sleep maintenance insomnia. However, doxepin, along with others in this list, particularly mirtazapine, are linked with significant weight gain. These antidepressants may show efficacy in patients with mood disorders and insomnia, but in addition to weight gain, the related sedating effect can be problematic for daytime functioning. Further, Everitt and colleagues found no evidence for amitriptyline or other antidepressants for long term treatment of insomnia [79].

### Trazadone vs. Hypnotics for Sleep

Trazadone is classified as an antidepressant, specifically a Serotonin-2 Antagonist-Reuptake Inhibitor (SARI). It has not been linked with weight gain, while many hypnotics have been. Yi and colleagues [81] completed a meta-analysis of trazadone for insomnia treatment, finding that it did not significantly improve sleep efficiency, sleep latency, total sleep time, or wake after sleep onset as compared to placebo, but participants reported improved sleep quality and reduced awakenings. The authors suggested that trazadone was likely effective for improving sleep maintenance by reducing early morning awakenings. Wichniak and colleagues [82] reported that trazadone was less effective than hypnotics for improving initial insomnia, but, as Yi and colleagues [81] also concluded, trazadone was more effective in treating sleep-maintenance insomnia. This was particularly true in patients with comorbid depression or other psychiatric disorders who were taking antidepressants with an activating effect.

### Antipsychotics as Insomnia Treatments: Effects on Weight

Antipsychotic medications have been used off-label with increasing frequency to treat insomnia and other disorders, such as anxiety and PTSD [83]. Quetiapine, a second-generation antipsychotic medication, has sedating effects and is not considered addictive or a drug of abuse, as hypnotics are. Quetiapine’s activity targets antihistamine and antiadrenergic action at lower doses (25–100 mg), causing sedation/hypnotic effects. Moderate doses also target serotonin (300–600 mg), thus improving mood, and high doses impact dopamine, in turn improving psychosis and deep sleep [83]. However, second generation anti-psychotics, including quetiapine, clozapine, and olanzapine, are known to cause weight gain and metabolic disease. Weight gain occurs in about two-thirds of patients, with mean gains between 5 and 10 pounds over the course of a year of quetiapine treatment at doses of 200 mg or less [83]. The exact mechanism for weight gain and metabolic dysregulation is unknown but may be linked to increased appetite from the antihistamine effect.

Given the risk with quetiapine for weight gain, metabolic dysregulation, and other adverse effects, such as akathisia, periodic leg movements, and rarely, tardive dyskinesia, its best use for insomnia treatment may be among patients seeking care for psychiatric disorders so that symptom relief may be optimized, instead of among persons without any clinically significant mood, anxiety, or psychotic symptoms.

## Conclusion

The research investigating the relationship between insomnia and obesity is inconclusive, however, recent research supports the notion that short sleep duration is associated with weight gain, particularly among children and adolescents. Among adults, weight gain may be driven more by greater sleep variability, later sleep timing, and poorer sleep quality. Future research should continue to identify and address mediators of the association between short sleep and weight gain across the lifespan.

There is mixed evidence that incorporating sleep goals into behavioral weight loss improves weight loss outcomes, particularly among adults. Stronger evidence has been seen among children and adolescents. A sleep extension intervention showed promise when nighttime sleep was extended over 1 h/night; however, future studies are urgently needed that include larger, more diverse samples with longer study durations and follow-up. The most common pharmacotherapies for sleep are not associated with weight change, but antidepressants and antipsychotics generally are. Continued efforts should be made to standardize the reporting of weight change for all pharmacotherapies to better inform patients.

## Data Availability

No datasets were generated or analysed during the current study.
